# Lymphoid stroma in all its states

**DOI:** 10.3389/fimmu.2025.1633235

**Published:** 2025-07-16

**Authors:** Nicolas Barbier, Valentin Isen, Karin Tarte, David Roulois

**Affiliations:** ^1^ UMR 1236, Univ Rennes, INSERM, Établissement Français du Sang, Équipe Labellisée Ligue contre le cancer, Rennes, France; ^2^ SITI laboratory, CHU Rennes, Établissement Français du Sang, Rennes, France

**Keywords:** lymphoid stromal cells, lymphoma, tumor draining lymph node, tertiary lymphoid structure, lymph node

## Abstract

Stromal cells are found in all tissues of the body. Among them, lymphoid stromal cells (LSCs) correspond to the cell subsets found in secondary and tertiary lymphoid organs. LSC heterogeneity has been characterized in depth in mice based on cell-fate mapping, high-resolution imaging and single-cell RNAseq analysis, and more recently in humans despite the difficulty of accessing these rare cell populations. At steady-state, LSCs organize discrete anatomical niches in lymphoid organs and orchestrate adaptive immune response. Studies of LSCs at the single cell level have identified a wide role for these cells in various pathological conditions, including solid tumors, autoimmune diseases, and lymphomas. In this review, we will discuss the diversity and plasticity of LSCs and LSC-like cells as well as their functions in pathological settings, with a focus on cancer and autoimmune diseases. Altogether, it highlights the importance of increasing our understanding of these cells, to use them as a target for novel therapeutic strategies.

## 1 Introduction

The stroma is a crucial part of the human organism. This compartment is essential for the function of the organs and forms the basic layer on which the specialized cells (called parenchymal cells) are embedded. Stromal cells are a highly heterogeneous subset of cells generally divided into three distinct groups (1). The first group consists of fibroblasts (producing the collagen-rich extracellular matrix and paracrine factors (2)), pericytes (regulating endothelial cells and remodeling blood vessels (3)) and telocytes (repairing, remodeling of the connective tissue, and tissue mechanical sensing (4, 5)), which are widely distributed. The second group is made up of stromal cells with proliferative and stem cell properties, such as bone marrow-derived mesenchymal stromal cells (MSCs) (1) or adipose-derived stromal cells (ASCs), depending on their tissue of origin (1, 6). The last type is organ-specific stromal cells, such as specialized lymphoid stromal cells (LSCs) found within secondary lymphoid organs (SLOs), specifically follicular dendritic cells (FDCs) and fibroblastic reticular cells (FRCs) (1, 7). The role of LSC in vaccination and in response to infection as antigen presenting cells and organizers of the lymphoid tissue dynamics has been well characterized. However, it is becoming increasingly clear that these cells are also key players in numerous pathological conditions and could be remodeled or modified to maintain or reduce the severity of inflammatory conditions. For instance, LSCs or cells presenting LSC characteristics are found in solid tumor draining lymph nodes (dLNs). These cells can also be found in tertiary lymphoid structures (TLSs) in cancers or autoimmune diseases, where they are called immunofibroblasts. In lymphomas these cells directly support tumor cells within invaded SLOs and eventually bone marrow (BM). They could be considered as the lymphoid counterpart of the cancer-associated fibroblasts (CAFs) which are found in solid tumors. This review focuses on LSCs in normal and pathological conditions, discussing the interest of considering these cells to develop new therapeutic approaches.

## 2 The lymphoid stroma in a physiological setting

The general structure of the 1,200 human LNs (8) is delimited by a border defined by a collagen capsule and organized into several specific intra-tissular compartments. The cortex contains mainly B cells (9) that are grouped in follicles whereas T cells are mostly located in the paracortex (9). Finally, the medullary zone is rich in myeloid and plasma cells. Extensive irrigation by blood vessels and afferent and efferent lymphatic vessels supports exchange of immune cells between the LNs and the circulation.

LNs can be divided into several areas, including the subcapsular sinus (SCS), the paracortex, which is enriched in T-cells, the cortex containing the B-cell follicles, where germinal centers (GCs) are induced during immune responses, and the medulla (10). Each of these areas contains distinct stromal cell populations.

In 2012, the ImmGen consortium identified the major types of lymphoid stromal cells (11) in mice. Among them, two endothelial cell populations were described (both positive for CD31/PECAM-1): blood endothelial cells (BECs), which are negative for podoplanin (PDPN, gp38 in mice) and gp38^+^CD31^+^ lymphatic endothelial cells (LECs). Three non-endothelial cell types were identified: gp38^-^CD31^-^ double negative cells (DNCs) and gp38^+^CD31^-^ LSCs including CD21/CD35-expressing FDCs, populating B-cell follicles, and CD21/CD35neg FRCs residing outside follicles. At least 3 populations of functionally and spatially distinct gp38^+^CD31^-^ FRCs were later described. First, RANKL^pos^ marginal reticular cells (MRCs), reside at the edge of the follicle underneath the SCS, and are involved in the delivery of small antigens to B cells. Second, medullary FRCs (MedRCs) form the major structural component of the plasma cell niche within LN medullary cords (12). Finally, FRCs located just around follicles were regularly called T-cell reticular cells (TRCs). Similarly, FDCs could be divided into dark zone (DZ) and light zone (LZ) FDCs depending on their localization within GCs. DZ-FDCs express high amounts of CXCL12 and are involved in the recruitment of CXCR4^hi^ centroblastic B cells in the DZ where they proliferate and accumulate random somatic mutations in the variable regions of immunoglobulin genes. LZ-FDCs retain intact antigens on their surface and contribute to the selection of high affinity centrocytes, that will compete to pick up antigens and present them as antigenic peptides to cognate T follicular helper cells (Tfh), which in turn provide them survival and differentiation signals.

These LSC subsets have been further characterized in humans (13–16). CD49a-expressing FRCs reside in the paracortex, support B and T cell survival and produce numerous cytokines and chemokines. The DNC is thought to be a heterogeneous population of perivascular cells (PvCs) including pericytes and mural cells. These cells contribute to the remodeling, stabilization and function of blood capillaries (17). With the advancement of single cell technologies, several studies in both mice and humans have revealed a high degree of heterogeneity of LSCs, allowing further refinement of the classification of these cell subtypes (18, 19). This has already been described in detail in high-quality reviews (10, 20–22). **Table 1** describes the different subtypes and their correspondence between mouse and human, including markers that are widely used to define these populations. The relationship between these markers and their specific functions in physiological and pathological conditions still needs to be elucidated. Briefly, TRCs could be subdivided into different populations. Two based on their expression of Ccl19 (high or low), and one expressing Cxcl9, the T/B border reticular cells (TBRCs) in proximity with the B cell follicle and the interfollicular reticular cells (IFRCs) close to the interfollicular regions (18, 23). Finally, scRNA-seq data in humans suggested that at least two populations of PvC exist based on ATG3 expression (15), however the exact role of these two populations remains unknown. A major common feature of FDCs and FRCs is that they derived from resident local mesenchymal precursors, including adventitial cells and adipocyte precursors and require both tumor necrosis factor-α (TNF) and lymphotoxin-α1β2 (LT) produced by immune cells for their maturation and maintenance as immunologically competent cells (20, 24). It has also been shown in mice that MRCs can serve as a precursor for FDCs, allowing their cellular renewal after infection (25). Few publications in humans suggest that PvCs have progenitor properties (13, 15). In addition, trajectory analysis, both in mice and human, suggests that adipocyte precursors expressing Pi16, the marker of universal fibroblasts (26), may be at the root of the LSC lineage (13, 15).

**Table 1:** Table of characteristics and markers of main human stromal populations.

Subset	Cells	Markers	Location	Alias	Function	Reference
FDC	Light zone Folicular dendritic cell (LZ-FDC)	PDPN^+^ (hs/mm) CD21^+^ (hs/mm) CD35^+^ (hs/mm) CXCL13^+^ (hs/mm) CXCL12^-^ (hs/mm)	GC light zone	FDC (before LZ-FDC) CXCL13-expressing reticular cell	Drive naive B cells and centrocytes to GC LZ via CXCL13 Orchestrate B cell clustering and follicle formation	(132) (hs) (19) (mm)
	Dark-zone Folicular dendritic cell (DZ-FDC) (not described in humans)	PDPN^+^ (mm) CD21^-^ (mm) CD35^-^ (mm) CXCL13^-^ (mm) CXCL12^+^ (mm)	GC dark zone	CXCL12-expressing reticular cell (CRC)	Drive centroblast to GC DZ via CXCL12	(133) (mm) (19) (mm)
Marginal reticular cell (MRC)	MRC	PDPN^+^ (hs/mm) CXCL13^+^ (hs/mm) RANKL^+^ (hs/mm) TNFSF11^+^ (hs/mm)	Subcapsular sinus		B cells guidance via CXCL13 Delivery of small antigens to B cells Potential FDC precursor	(134) (hs) (135) (hs) (136) (mm) (19) (mm)
perivascular cell (PvC)	PvC ATF3^low^	PDPN^-^ (hs/mm) CD49a^+^ (hs) ACTA2^low^ (hs)	Near blood vessels	Perivascular reticular cell (PRC) Double negative cell (DNC) Smooth muscle cell (SMC) Pericyte Mural cell	Potential adult FRC precursor	(13) (hs) (15) (hs) (19) (mm)
	PvC ATF3^high^	PDPN^-^ (hs/mm) CD49a^+^ (hs) ACTA2^High^ (hs)				
T cell zone reticular cell (TRC)	T–B border reticular cell(TBRC)	PDPN^+^ (hs/mm) CCL19^low^ (hs/mm) CCL21^+^ (hs/mm) Gremlin1^+^ (hs/mm)	T–B border		Support conventional DC survival, T and B cell interactions	(137) (hs) (19) (mm)
	TRC CCL19^high^	PDPN^+^ (hs/mm) CCL19^high^ (hs/mm) CCL21^+^ (hs/mm)	Paracortex		Drive T cells, B cell and DC migration, via CCL19, CCL21and CXCL12	(15) (hs) (133) (mm) (19) (mm)
	TRC CCL19^low^	PDPN^+^ (hs/mm) CCL19^low^ (hs/mm) CCL21^+^ (hs/mm)	Follicle, interfollicular region and paracortex		Drive B cell and DC migration	(137) (hs) (19) (mm)
	CXCL9 TRC	PDPN^+^ (hs/mm) CCL19^+^ (hs/mm) CCL21^+^ (hs/mm) CXCL9^+^ (hs/mm)	Interfollicular region		Support DC, T and B cell interactions	(137) (hs) (19) (mm)
Medulla reticular cell (MedRC)	MedRC	IL-6^+^ (hs/mm) BAFF^+^ (hs/mm) CXCL12^+^ (hs/mm) APRIL^+^ (hs/mm) CXCL13^-^ (hs/mm)	Medulla		Plasma cell survival via APRIL, IL-6, BAFF	(12) (hs)

Human lymph node stromal cells are isolated by absence of main endothelial and erythrocyte marker CD31/PCAM1 and absence of lymphoid and myeloid marker CD45/PTPRC. Table shows the markers for homo sapiens described population (hs) and corresponding markers found in mus musculus (mm). For a more complete description of markers in mice, please refer to reviews (21, 138, 139).

## 3 Stromal cell remodeling in draining lymph nodes from solid tumors

### 3.1 Historical definition of CAF in solid tumor context

The heterogeneity and importance of CAFs was first described in solid tumors. A study in pancreatic ductal adenocarcinoma (PDAC) first identified two majors distinct CAF populations: inflammatory CAFs (iCAFs spatially distant from the tumor) and myofibroblast-like CAFs (myCAFs close to the tumor) (27). The two populations are closely related; the IL1/NF-κB and JAK/STAT pathway induce the iCAF phenotype, which is blocked by the TGFβ pathway to induce a myCAF phenotype in PDAC (28). It has also been suggested that an intermediary exists between iCAF and myCAF that may be interconvertible, depending on the location and the signals received (28), but further research is needed to confirm this claim. Using a newly developed type of clustering in scRNA-seq, similar populations have also been identified in colorectal cancer, namely CAF-B, with a myofibroblast profile and CAF-A with a more intermediate phenotype expressing FAP and extracellular matrix remodeling molecules (29). Subsequent research has deepened our understanding of the functional role of myCAFs and iCAFs, and has added a third class of CAFs, called antigen-presenting CAFs (apCAFs), discovered in 2019 within human and mouse PDAC scRNA-seq data (30) and able to activate CD4 T cells *in vitro* (30, 31).

A recent multiomic study of several solid tumors in mice (breast) and human (breast, skin, and pancreas) has proposed three spatially distinct and conserved populations of CAFs (32): steady state like, mechanoresponsive, and immunomodulatory CAFs. The proportions of these cell subclusters vary in response to mechanical force and immunotherapy, affecting cancer growth (32). It is tempting to speculate that these populations correspond to the myCAF/iCAF/apCAF classification described in PDAC, a hypothesis supported by another analysis of CAF heterogeneity in breast cancer (33). An atlas of microenvironment cells across 226 samples from 10 solid tumor types revealed a similar classification of CAF (34). In this study, all fibroblasts clustered according to their subtype independently of the tumor of origin, and three distinct subtypes were identified, termed myofibroblast (CAF_myo_), inflammatory (CAF_infla_) and antigen-presenting (CAF_ap_) (34) which closely correspond to the classical myCAF/iCAF/apCAF. Other CAF types are identified, such as adipogenic CAF (CAF_adi_), potentially derived from vascular ASCs and identified *in silico* in pancreatic, lung, head and neck, ovarian and breast cancers (35). Other minor CAF subtypes identified include endothelial-to-mesenchymal transition CAF (CAF_EndMT_) and peripheral nerve-like CAF (CAF_PN_) (34). In solid tumors, all these CAF populations could influence the course of the disease and carry pro-tumoral properties which are extensively reviewed elsewhere (36, 37).

### 3.2 Mechanisms for dLN invasions by solid tumors

Human cancers metastasize to distant organs through the blood and lymphatic systems. In many cancers, such as melanoma and breast cancer, dLNs are the preferred tissue targets. In fact, dLN is the first site to be drained by the primary tumor, which then allows for transport of tumor cells along the lymphatic system (38). In many cancers, invasion of the dLN by tumor cells is assessed at diagnosis and is associated with poor patient prognosis. For example, in lung cancer, the presence of multiple metastatic nodes has a negative impact on patient prognosis and survival (39).

**Figure 1:**
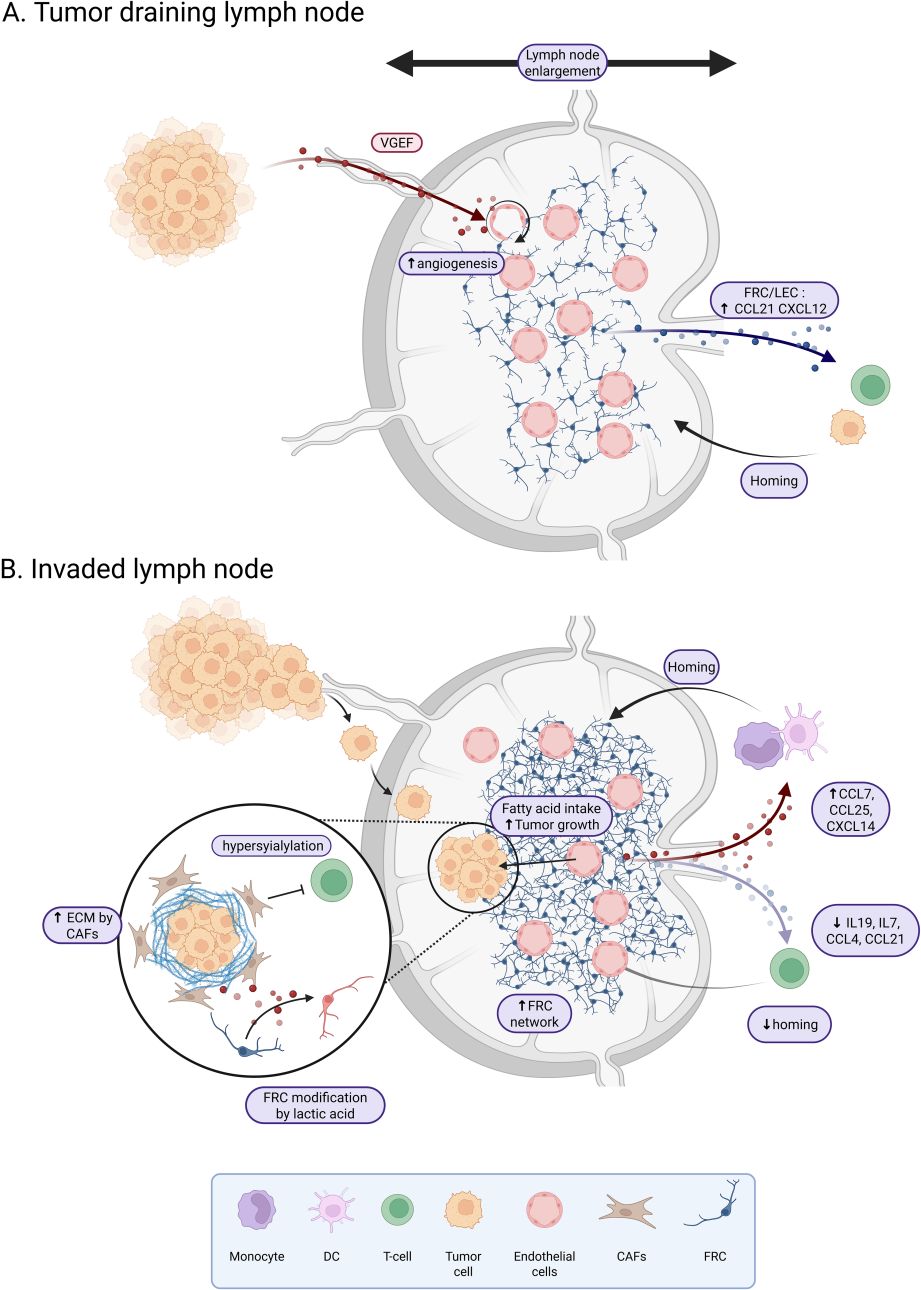
Tumor draining lymph node reshaping before and after tumor invasion. **(A)** Non-invaded tumor dLNs are characterized by an enlargement associated with an active angiogenesis due to VEGF production by the tumor. LECs and FRCs from tumor draining lymph nodes show an increased expression of CCL21 and CXCL12 which favor the homing of T-cells and tumor cells. **(B)** In invaded dLN, the FRC network is dense. Stromal cells exhibit an increase in CCL7, CCL25 and CXCL14 which favor the homing of monocytes and dendritic cells, and a decrease in IL19, IL7, CCL4 and CCL21 which reduce the homing of T-cells. LSCs of the dLN are hyper-sialylated and produce extracellular matrix leading to the inhibition of T-cell response and infiltration. Tumor invasion will also affect the metabolic landscape with fatty acid uptake to support tumor growth and a reprogramming of FRCs by tumor-produced lactic acid. Created in BioRender.

Tumor cells will first colonize the subcapsular sinus of the dLN, then invade the cortex, and grow until the entire LN is completely replaced by the tumor (40). However, dLN already exhibits a specific phenotype before tumor infiltration, such as enlargement compared to steady state situation (41) (**Figure 1A**). This enlargement may result from an enrichment of functional blood and lymphatic vessels (41, 42) associated with an increased vascularization of non-metastatic dLN compared to non-dLN. Nascent vessels develop from endothelial cells derived from high endothelial venules (HEVs), whose proliferation increases significantly (41). This pre-metastatic stage, first described in 2006 by Kaplan et al. (43), is initiated by several factors, including VEGF-A and VEGF-C. VEGF-A is overexpressed by primary tumors and has been shown to play a role in the pre-metastatic lymphangiogenesis of dLNs (44). Unlike VEGF-A, VEGF-C does not increase the growth of primary tumor but induces lymphatic vessel hyperplasia and promotes tumor metastasis in dLNs (45). In PDAC, CD44 expressing extracellular vesicles released by the primary tumor enables the establishment of the pre-metastatic niche in both dLN and lung (46). Similarly, in melanoma, tumor cells have been shown to produce the heparin-binding factor midkine, which leads to paracrine activation of the mTOR pathway in LECs and subsequent expression of VEGFR3, which is not normally expressed in adult LECs, suggesting a functional role for midkine in neo-lymphangiogenesis (47). Notably, high nodal midkine expression is associated with poorer disease-free survival in patients. Thus, primary tumors may somehow prepare LNs for metastasis by producing lymphangiogenic factors that enable the transport of malignant cells to dLNs. In fact, remodeling of LN endothelial cells may also promote tumor cell recruitment. In a healthy context, CCL21 is produced by LECs and FRCs and regulates the homing of naive T cells and mature dendritic cells expressing CCR7. CCL21 produced by LECs has been shown to attract CCR7^+^ melanoma cancer stem cells (CSCs) in both mice (48) and humans (49). Furthermore, CXCL12 expressed by tumor-activated LECs in both axillary LN and lung can also attract CXCR4-expressing melanoma CSCs, thereby promoting metastatic growth (50). Similarly, in breast cancer, tumor cells that metastasize to dLN, bone marrow, lung and liver express both CCR7 and CXCR4 (51). Interestingly, LN enlargement is also observed during immune response. This phenomenon is controlled by PDPN+ FRCs that support LN stiffness and reduce their contractility in contact with dendritic cells (DCs) expressing the PDPN receptor CLEC2 (52, 53). It has been shown in a mice model of melanoma, that dedifferentiated melanoma cells produce IL-1, that inhibits the JAK1-STAT3 dependent contractility of FRCs, favorizing the establishment of a pre-metastatic niche (54).

### 3.3 Tumor dLN remodeling alter immune functions

dLNs are essential sites for the development of an adaptive immune response and play a crucial role in the response to immunotherapy such as immune checkpoint blockade (55, 56). The remodeling of dLNs upon metastasis impairs LNs immune functions (**Figure 1B**). This has been shown particularly in melanoma, where dLNs have reduced immune function, mainly due to immunosuppressive factors released by melanoma cells that induce local dLN paralysis and prevent recognition of melanoma cell antigens by DCs (57). A similar effect has been observed in breast cancer dLNs (58). This effect could be explained by HEVs extensive changes and remodeling, including vascular dilation, thinning of the endothelium, as well as loss of CCL21 expression by perivascular stromal cells in association with HEV dysregulation, affecting the recruitment of T cells to the dLN (59–61). Indeed, after adhesion to HEV, lymphocyte recruitment in the LN and extravascular migration in the paracortex requires surface-bound, unlike soluble, CCL21 (62). Immobilization of CCL21 depends on the binding of the C-terminus of CCL21 to the extracellular matrix (ECM) and to sulfated glycosaminoglycans on the cell surface, including heparan sulfate (63). Taken together, these observations highlight the importance of the dLN and the potential priming of the dLN niche, specifically the remodeling of endothelial cells prior to tumor cell invasion. In addition, dysregulation of the endothelial network could be increased by the depletion of smooth muscle cells surrounding blood vessels and an increased expression of CXCL12 by the remaining cells, favoring activation of specific inflammatory pathways as shown in dLNs of esophageal squamous cell carcinoma (64).

The LSC compartment is another player that could be extensively remodeled in dLN. A few studies have described transcriptional reprogramming of LSC upon dLN metastasis. In a mouse model of melanoma, FRCs proliferate in response to tumor cell signals and this proliferation is associated with a remodeling of the FRC network (59). In this study, a kinetic transcriptomic profiling of FRCs from dLN versus non-dLN allows to identify deregulation of pathways involved in matrix remodeling and immune function, with a decrease of *Il19*, *Il7*, *Ccl4* and *Ccl21* in dLNs, associated with a reduction of T cell recruitment. In parallel, the increased expression of *Ccl7*, *Ccl25* and *Cxcl14* favors recruitment of monocytes and DCs. Collectively, these data argue for the role of FRC remodeling in the establishment of an immunosuppressive microenvironment.

In solid tumors, CAFs are known to produce several matrix metalloproteinases that allow remodeling of the tumor microenvironment (TME) (65) and prevent immune cell entry into the tumor site (66). In LN, FRCs are known to produce ECM, whose composition is affected during LN expansion following inflammation (67). However, whether these mechanisms are deregulated in dLNs remains unknown. In colorectal cancer, LSCs exhibit an hypersialylation of glycans (68) and this hypersialylation reduces the anti-tumor activity of CD8^+^ T cells. Sialoglycans are recognized by siglec (sialic acid-binding immunoglobulin-type lectins) expressed on the surface of immune cells (69) and regulate their functions. Overall, hypersyialylation leads to immune evasion, drug resistance, tumor invasion, and metastasis (70–72). Another immunosuppressive mechanism involving FRCs has been described in colon and bladder cancer, where FRCs produce hyaluronan, which directly interacts with PD-L1^+^ FA/80^+^ macrophages involved in immune escape and resistance to therapy (73). In cervical cancer, the ratio of tumor-to-stroma CD8^+^ T cells is negatively correlated with CAF density, but also with LN metastases. However, whether CAF density in tumors affects dLN stromal cell identity has not been evaluated yet (74). A comparative study of CAFs from tumor site and fibroblasts from the dLN in oral squamous cell carcinoma suggests that resident FRCs in the dLN differentiate into tumor CAF-like cells, with dysregulation of collagen matrix genes, reduced crosstalk with immune cells and association with infiltration of metastatic cells beyond the dLN capsule, which may favor distant metastasis and raises the interest to better study fibroblasts in the dLN as a new therapeutic option (75).

Finally, the metabolic environment is also affected in dLN from solid tumors with a potential impact on immune cells. Particularly, single cell analysis of immune and stromal cells in a mouse model of breast cancer revealed that dLN FRCs have a different metabolic and transcriptomic profile compared to FRCs from tumor-free LNs. dLN FRCs accentuate oxidative phosphorylation and fatty acid metabolism, which may contribute to the dLN metabolic switch and tumor metastasis (76). Fatty acid oxidation is mediated by a transcriptional coactivator, YAP, which is activated in FRCs following the accumulation of high levels of bile acids in dLN (71). In another study, lactic acid was shown to be released by tumor cells in the dLN, leading to the reprogramming of FRCs with an increase in Pdpn and Thy1 and a decrease in IL7 expression. These transcriptomic and proteomic changes are associated with a decrease in FRC intracellular pH by lactic acid and an alteration of mitochondrial functions (77). Interestingly, the activity of T cells is affected by metabolic changes. Lactic acid has indeed be shown to hinder antitumor T cell responses by increasing regulatory T cells, and decreasing both CD8 T cells and IFNγ+ T cells (78).These studies suggest that FRCs in dLNs may remodel the niche through metabolic reprogramming.

In summary, FRCs are involved in many immune functions that might be deregulated in dLN. Most studies on dLN have been carried out in mice, and to date, due to the paucity of stromal cells and the ethical concerns associated with harvesting dLN for research purposes, almost no studies have described the remodeling of these cells in humans. Given the importance of these stromal cells for LN function, there should be increased interest in characterizing these cells in humans in the near future.

## 4 The role of lymphoid stroma in tertiary lymphoid structures

**Figure 2:**
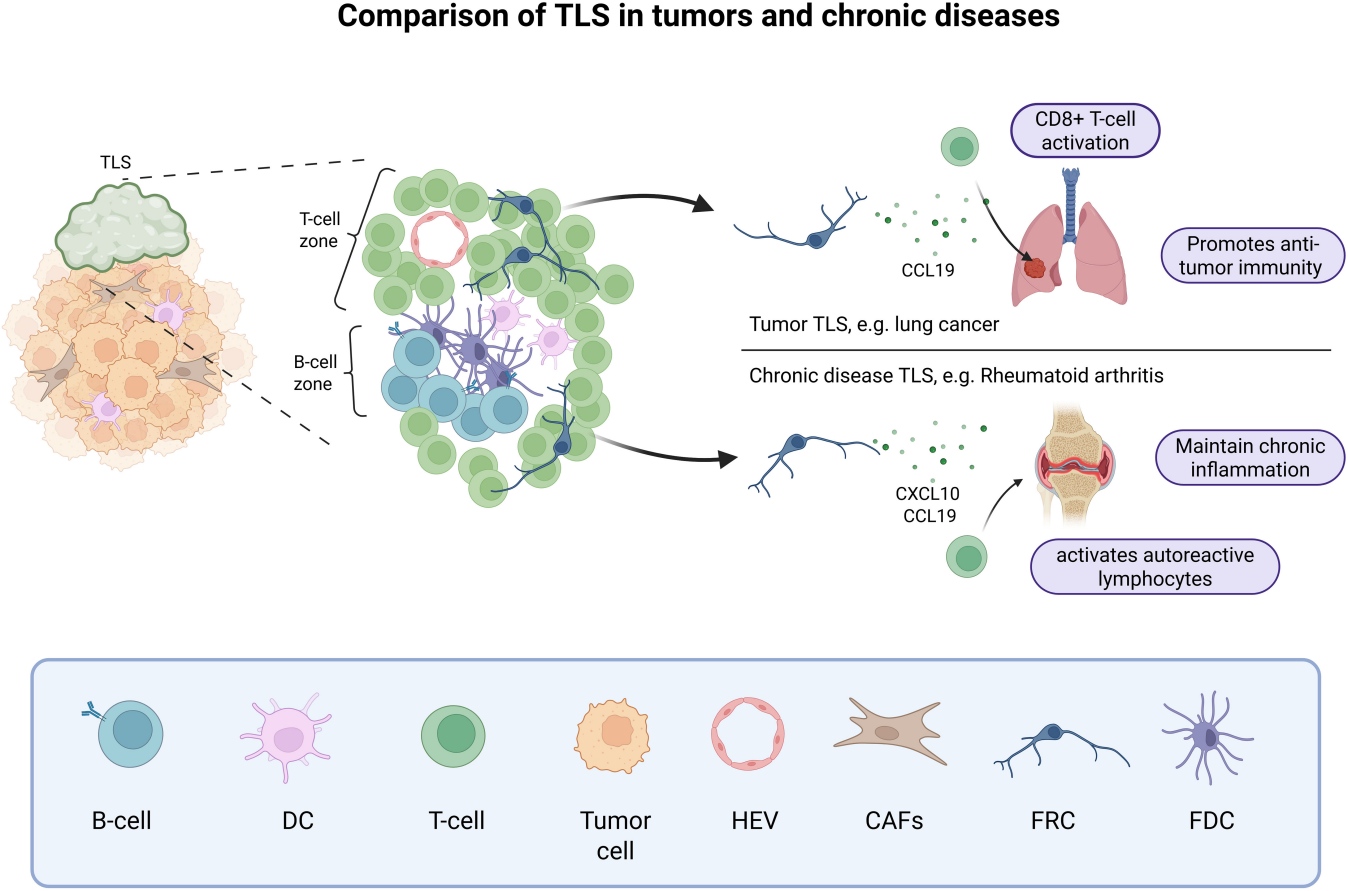
Tertiary lymphoid structures in tumors and chronic diseases. In tumor and chronic diseases, B cells and T cells can form aggregates called TLSs, that mimic a secondary lymphoid organ. These TLSs are usually close to the active immune hotspot and can promote anti-tumor immunity through CCL19 production by FRCs and CD8+ T-cell activation or maintain chronic inflammation through stromal cell production of CXCL10 and CCL19 and the recruitment of autoreactive lymphocytes. Created in BioRender.

In pathological inflammatory conditions, B and T cells can aggregate and form structures that resemble LNs, with discrete T-cell and B-cell zones, the latter eventually containing GCs at different states of maturation. These structures are called tertiary lymphoid structures (TLSs). Although TLSs have much in common with LNs, they are not distinct organs; they are variable in structure, have no defined location, and are generally not encapsulated (79). They are defined as ectopic structures that develop postnatally in non-lymphoid tissues. TLSs contain specialized fibroblasts presenting characteristics of classical LSCs, such as FRCs and FDCs (80, 81) (**Figure 2**). It has been described that TLSs occur in cases of chronic inflammation, such as autoimmune diseases, chronic infections or tumors (79). They create a favorable environment for local eradication of infections and have also been associated with improved outcomes in cancer immunotherapy (82, 83). An important study demonstrated that the presence of TLSs in a mouse model of lung cancer is not sufficient to eliminate the tumor, but that suppression of regulatory T cells (T_regs_) enables activation of anti-tumor immune cells in tumor TLSs and drives tumor destruction (84). However, in autoimmune diseases, TLSs contribute to the maintenance of aberrant inflammation and serve as an activation site for autoreactive lymphocytes (85), with a potential role for fibroblasts in the selection of these cells (86). In murine non-lymphoid tissues, local fibroblasts can acquire LSC phenotype and function during chronic inflammation, becoming immunofibroblasts expressing adhesion molecules, lymphoid chemokines, and lymphocyte survival factors classically produced by LSCs (87). Interestingly, immunofibroblasts and their precursor cells are necessary for the induction of TLSs and represent an interesting target in autoimmune diseases. In intraperitoneal tumors, CAFs themselves can be polarized into organizer cells that orchestrate TLSs development with expression of LSC genes such as *PDPN*, *CXCL13*, *BAFF* and *APRIL* (88). Thus, the origin of the stromal cells in TLSs is therefore different from that of SLO LSCs, which are of embryonic origin (89). Another origin has been proposed in lung cancers where perivascular precursors (both mural and adventitial cells) could differentiate into CCL10-expressing TLS FRCs, promoting CD8+ T cell antitumor activity (90). A study suggests that sustained interferon and antigen recognition are necessary for the induction of TLSs in the lung independently of FAP+ fibroblasts and associated with CCL19 production independently of LTβR signaling, highlighting another mechanisms of TLSs induction (91). Interestingly, the presence of CCL19+ fibroblast was also found associated with immune cell infiltration in hepatocellular carcinoma (92). Overall, these studies highlight the therapeutic interest for a better understanding of the pathways that support the antitumor properties of TLS stromal cells. Future studies should determine whether CAFs and immunofibroblasts could share a common precursor cell, this would help to identify pathways to reprogram CAFs into immunofibroblasts.

The formation of TLSs in chronic diseases can be associated with both a good and a bad prognosis, depending on the pathology. Therefore, a fine understanding of the mechanisms involved in the development of these structures, as well as in the heterogeneity and function of TLS fibroblasts is essential to open the possibility of stromal cell-oriented immunotherapeutic strategies (93, 94). The heterogeneity of immunofibroblasts in autoimmune diseases has been studied in synovium, intestine, lung, and salivary glands identifying two clusters of immunofibroblasts shared by different diseases (95), A first cluster of CXCL10^+^ CCL19^+^ fibroblasts interacts with immune cells and produce CCL2, which could be a target of epigenetic drugs, as proposed by our group (96). This FRC-like fibroblast cluster is particularly present in Sjögren’s disease associated with TLS induction. The second cluster is defined by SPARC and COL3A1 expression and shows an enrichment in ECM binding factors. These cells are localized near the vessels and are closely associated with the PvC phenotype found in LNs. Interestingly, another scRNA-seq study in Sjögren’s disease describes that PvCs or mural cells are immunocompetent cells, producing the CCL19 and CCL21 chemokines necessary for T cell recruitment and organization in TLSs (97). Fibroblast-like synoviocytes (FLSs) with LSC-like phenotype have also been described in rheumatoid arthritis. FLSs interact with immune cells and support the formation of ectopic lymphoid-like structures contributing to pathogenic immune response in synovial tissue. Their ability to express LTβR, IL-7, RANKL, CXCL13, CXCL12, CXCL21, CXCL19, VCAM-1, ICAM-1, and PDPN brings them closer to the lymphoid tissue organizer phenotype (98–100). It is interesting to note that they also express BAFF, APRIL, which can give survival signal to autoreactive B cells (101). Thus, blocking their activities could reduce inflammation.

However, these studies do not address the question of fibroblast heterogeneity in tumor TLSs, nor whether these TLS stromal cells share similarities or differences with extra-TLS solid cancer CAFs and CAFs found in hematological malignancies.

## 5 Lymphoid stromal cells in hematological tumors

All of the previous studies were performed in solid tumors. We could therefore ask whether similar heterogeneity is observed in hematological malignancies where the primary site is the LN. The World Health Organization (WHO) classifies hematolymphoid tumors into two families of cancers called lymphoid neoplasms and myeloid neoplasms based on the primary cell subtype (102). Cancers that arise from the myeloid or lymphoid lineages are termed leukemia when they arise in the bone marrow. Cancers are classified as lymphoma when they arise in the lymphoid tissues system (i.e., lymph nodes and tissues) (102) and TME has been described as an important contributor to lymphoma development (103).

Mature B-cell malignancies include a large panel of hematological malignancies whose tumor clone develops from mature B cells within lymphoid tissues. Among them, follicular lymphoma (FL) and diffuse large B-cell lymphoma (DLBCL) are the two most common lymphomas and result from the transformation of GC/post-GC B cells. Stromal cells have been identified as key drivers of FL pathogenesis (104), both in LNs and in invaded bone marrow (BM) where ectopic LSCs, including FRC and FDC-like cells, are locally induced from uncharacterized LSC precursors in contact with malignant B cells (105–107). FL-B cell-derived extracellular vesicles can even prime BM stromal cells at distance thought the TGFb pathway, favoring anchorage of FL-B cells (107), a mechanism that mimic the one observed in solid tumors with tumor cells priming dLN for subsequent invasion. LN LSCs are remodeled by FL-B cells under the influence of TNFa, LTa1b2, and TGFb (13, 15), but also by IL-4 overexpressed by FL-Tfh cells (105, 108). How these signals are integrated by LSC/LSC precursors remains to be elucidated. Lymphoid CAFs in FL provide critical signals that directly promote tumor B-cell growth and contribute to the building and function of the tumor-supportive niche, particularly through the overexpression of CCL2, CXCL12, CCL19, and CCL21 and the modification of extracellular matrix composition and organization (13, 105, 106, 109, 110) influences the tumor niche. Gain-of-function mutations of EZH2 are associated with an increased FDC network at the premalignant stage (111). Conversely, loss-of-function mutations of HVEM/TNFRSF14 alleviate an inhibitory signal deliver to BTLA-expressing Tfh, leading to an overexpression of TNFα and LTα1β2, and to a resulting activation of FRCs and FDCs (112). One study suggested that ERα^high^ FDCs were associated with a better FL patient prognosis (113). In contrast, extensive remodeling of the lymphoid CAFs compartment, with expansion of desmin+ vimentin+ fibroblasts and upregulation of ECM genes has been shown to be associated with early relapse and poorer outcome in FL patients (114).

In 30% of cases, FL progresses to aggressive DLBCL. In DLBCL, lymphoid CAFs have been shown to play an important role in patient clinical outcomes. Indeed, transcriptomic signatures reflecting matrix deposition and immune cell infiltration have been associated with a better overall survival, in contrast to a signature associated with angiogenesis (115). This classification has been further refined with the identification of 4 subtypes in DLBCL, with GC-like TME associated with a better prognosis than TME-depleted microenvironmental cells (116). In a more recent study, numerous tumor ecotypes have been associated with patient survival and response to therapy, highlighting another level of complexity (117). Interestingly, evidence is accumulating for the critical role of vascular remodeling, particularly in high-grade lymphoma (118). In classical inflammatory conditions, vascular remodeling is highly dependent on FRCs, which produce vascular endothelial growth factor-A (VEGF-A) under cytokine stimulation (118). However, in high-grade B-cell lymphoma, FRCs VEGF-A production is bypassed and produced by lymphoma B cells to recruit primarily HEV cells (118). Conversely to lymphoid CAFs in FL, DLBCL lymphoid CAFs exhibit a decreased production of CCL19/CCL21 lymphoid chemokines and overexpress PD-L1 thus reducing T-cell recruitment and suppressing T-cell function (119). This lymphoid CAFs dysfunction could hinder the efficacy of CAR T-cells and other immunotherapeutic strategies in DLBCL. A clinical trial evaluating CAR-T therapy on LBCL (large B-cell lymphoma, regrouping several lymphoma subtypes including DLBCL) reported that a signature of immunosuppressive TME is associated with a negative outcome (120). The author supposed that tumors with this gene expression signature containing myeloid, stromal and endothelial genes, hypoxia response genes and TGF-β genes, display a reduced infiltration of immune cells (120).

Excluding FL and DLBCL, lymphoma TME is still poorly described. In Burkitt lymphoma, more than 90% of the cells present in this non-Hodgkin’s lymphoma are tumor cells, and the proportion of lymphoid CAFs is very low (103). Macrophages may play a role in the development of the disease, but there is no evidence of stromal cell involvement (121). Malignant cells have genetic aberrations which give them signals for survival and proliferation, eliminating the need for microenvironment stimuli (122). Like lymphoid CAFs in FL, stromal cells protect mantle cell lymphoma B-cells from apoptosis, promote their growth and increase resistance to treatment (123), notably by secreting BAFF (124).

The stromal microenvironment also plays an important role in chronic lymphocytic leukemia (CLL). CXCL12-expressing CLL-FDCs (e.g Dark-zone FDCs) have been shown to be required for chemotaxis and survival of indolent chronic lymphocytic leukemia (125), particularly through the production of BAFF (126). In addition, tumor B cells directly induce aberrant CXCL13 expression in non-FDC infiltrating stromal cells through LTβ receptor activation and retinoic acid signaling (125, 127). Lymphoid CAFs can also bind directly to CLL cells through their adhesion molecule VCAM-1, leading to retention of CLL cells (128). As in FL, BM stromal cells can produce extracellular vesicles that transport lncRNA, miRNA and cytokines and provide survival signals (98, 129).

**Figure 3:**
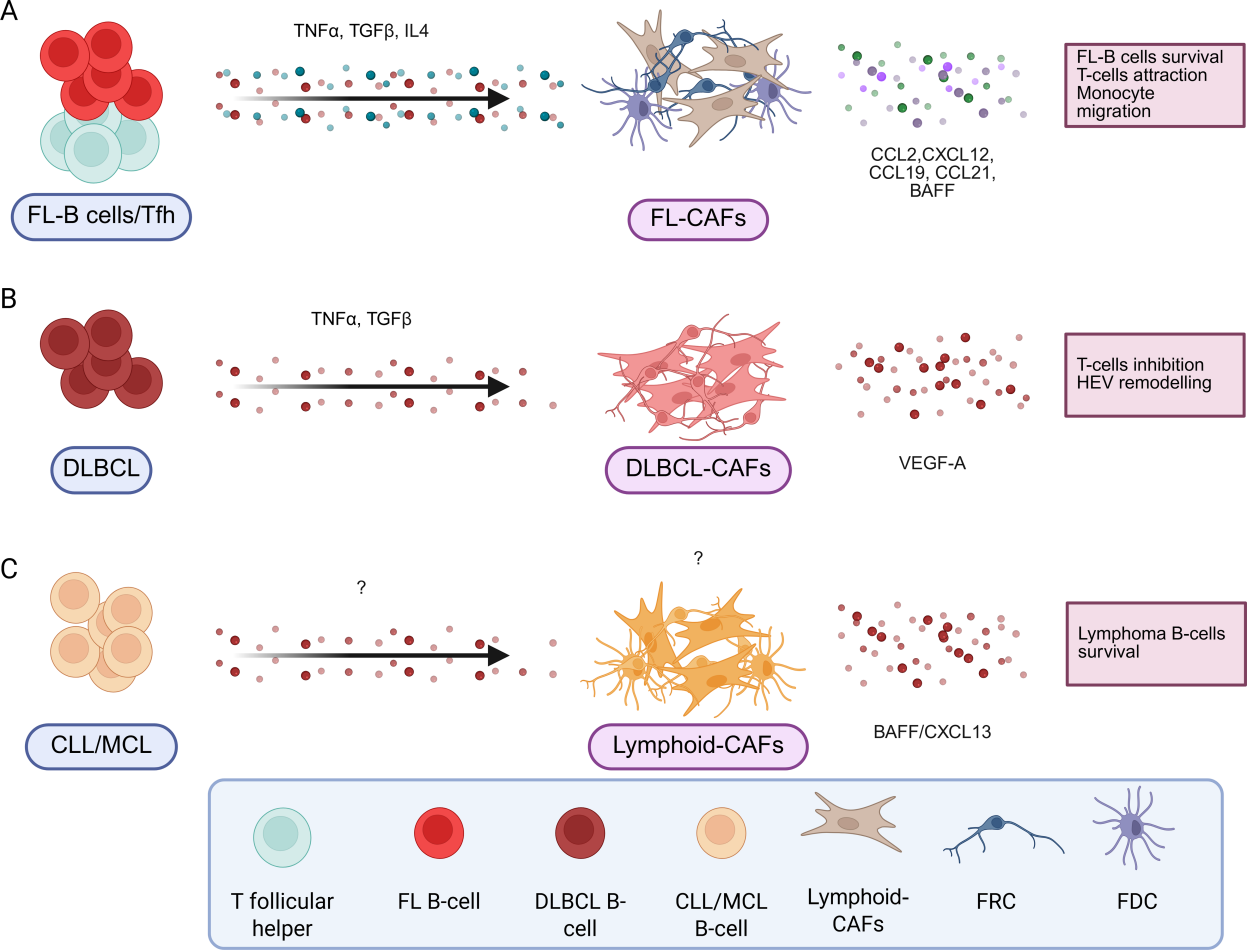
B-cell lymphoma induces pro-tumoral stromal cells with different signals. In B-cell lymphomas, different signals produced by the B-cell lymphoma but also by other cells present in the tumor microenvironment will induce pro-tumoral stromal cells by different mechanisms. They are resumed in the caption for FL **(A)**, DLBCL **(B)** and CLL/MCL **(C)**. Created in BioRender.

Overall, our knowledge of lymphoid CAFs in lymphoma is rather limited (**Figure 3**). It is not clear whether these cells exhibit the same heterogeneity as CAF from solid tumors, However, understanding how to target these cells to restore or enhance response to immunotherapeutic strategies should be a research priority in the future.

## 6 Conclusion and perspectives on future research

As described in this review, LSCs play an important role in the physiological function of SLOs. In a pathological context, in some cases they provide essential support for pathological development. Blocking or enhancing the development of these cells could open new therapeutic perspectives. For example, in breast cancer, the use of an anti-CD73 significantly reduces the immunosuppressive function of CAFs (130). Similarly, in melanoma, reprogramming of fibroblasts by a viral vector encoding an autoantigen could locally support the activity of T lymphocytes targeting the tumor (131). With the increased interest in CAFs in recent years, these examples of CAFs/lymphoid CAF targeting in solid tumors and hematologic malignancies will likely increase in the future.

Indeed, given the importance of stroma in the development of hematologic tumors, it is critical to further investigate stromal cell heterogeneity. Do all hematologic tumors exhibit the same lymphoid CAF heterogeneity? What is the lymphoid CAFs specificity and exact functional role of each tumor subtype? Does lymphoid CAFs heterogeneity allow to identify and develop a reliable and effective therapy against pro-tumoral lymphoid CAFs subtypes? Does lymphoid neoplasms with minimal stromal cells involvement like Burkitt lymphoma would beneficiate of such strategies? All of these questions would need to be addressed in the future to deepen our understanding of lymphoid CAF involvement and further improve patient standard of care.

In addition, to develop multi-cancer therapeutics, it is also essential to understand the relationships between solid and hematological cancer CAFs. Lymphoid CAFs are still understudied and comparisons between already known CAFs from PDAC and breast cancer are critical to assess the efficacy of drugs targeting pro-tumoral stromal cells. It will be important to determine whether therapeutic strategies targeting CAFs in both solid tumors and lymphoid neoplasms could be shared. This would require to assess if solid tumor CAFs and lymphoid CAFs share similarities, and to evaluate the specificity of each tumor type. Finally, it may be interesting to compare lymphoid CAFs from hematological tumors, with immunofibroblasts found in tumor TLSs and LSCs in dLNs, as these cells may play an ambivalent role in disease progression. Deepening our knowledge of LSCs in normal and pathological settings should allow us to develop targeted therapies against these populations in numerous diseases and sites.

Nevertheless, this review has critical limitations that must be addressed in future studies. First, most human studies rely on a small number of samples (n = 1 or 2) due to ethical and sampling challenges. Second, many studies use only murine models to gain mechanistic insight without validating their results on human samples. To improve the reliability of their findings, future studies should be conducted on human samples and analyze a larger number of samples.
